# Supplementary material for: Low husband involvement in maternal and child health services and intimate partner violence increases the odds of postpartum depression in northwest Ethiopia: A community-based study
Source: PLoS One. 2022 Oct 26;17(10):e0276809. doi: 10.1371/journal.pone.0276809 (PMC9604988; doi:10.1371/journal.pone.0276809)
Supplement: S1 File — (DOCX) [file pone.0276809.s001.docx]

English version of the questionnaire

Part I: Socio-demographic characteristics

| N.O | Questionnaire | Alternative choice for response | Skip code |
| --- | --- | --- | --- |
| 101 | How old are you? | (In years) |  |
| 102 | What is your educational level? | 1. Can’t read and write  2. Can read and write  3. Primary  4. Secondary  5. Diploma and above |  |
| 103 | What is your marital status? | 1. Single  2. Married  3. Divorced  4. Widowed  5. Separated |  |
| 104 | What is your occupation? | 1.House wife  2. Merchant  3. Government employee  4. self employed  5. Student  6. Others specify…………………………. |  |
| 105 | What is your religion? | 1. Orthodox Christian  2. Muslim  3. Protestant  4. Catholic  5. Other specify…………………………. |  |
| 106 | How much is the average monthly income of the family? | (In Ethiopian birr) |  |
| 107 | What is your husband educational level? | 1. Can’t read and write  2. Can read and write  3. Primary  4. Secondary  5. Diploma and above |  |
| 108 | What is your husband occupation? | 1. Daily labor  2. Merchant  3. Government employee  4. self employed  5. Student  6. Others specify…………………………. |  |

**Part II: Obstetric and medical characteristics**

| NO | Questioners | Alternative choices for response | Skip code |
| --- | --- | --- | --- |
| 201 | How many times did you give birth after 7 months? |  |  |
| 202 | Did you have ANC follow-up in your most recent pregnancy? | 1. Yes  2. No | If yes got to 203 |
| 203 | How many ANC visits did you had on your most recent pregnancy? | 1. Once  2. Twice  3. Three times  4. Four and above |  |
| 204 | Did you get information on mental health problems during pregnancy? | 1.Yes  2. No | If no got to 206 |
| 205 | Where did you deliver your most recent child? | 1. 1. At home 2. 2. At health post 3. 3. At health center   4. At government hospital  5. At private clinic/hospital |  |
| 206 | Did you have PNC visit in your most recent child? | 1. 1. Yes 2. 2. No | If no go to 212 |
| 207 | How many PNC visits did you have? | 1. 1. One 2. 2. Two 3. 3. Three or more |  |
| 208 | Known medical disorder present | 1. 1. Yes 2. 2. No |  |
| 209 | Known psychiatric problem present | 1. 1. Yes 2. 2. No |  |
| 210 | Mode of delivery | 1. 1. Cesarean section 2. 2. Spontaneous vaginal delivery |  |
| 211 | Was the last pregnancy planned? | - - - 1. Yes       2. No |  |
| 212 | Was your last pregnancy support by you husband or other families? | Yes  No |  |
| 213 | Have you had experience of adverse life events such as death of close family, husband, and friend? | 1. Yes  2. No |  |
| 214 | Husband/Partner involvement related questions on MNCH services | | |
| I | Did your husband go with you for ANC follow up at least once in your most recent pregnancy? | 1. Yes  2. No |  |
| II | Did your husband provide transport/gave money for transport during your recent pregnancy or delivery? | 1.Yes  2. No |  |
| III | Did your husband accompany to the hospital during labor for your recent delivery? | 1. Yes  2. No |  |
| IV | Did your husband discuss with health care providers during your recent pregnancy or delivery? | 1. Yes  2. No |  |
| V | Did your husband look after the child at home/stay with babies while you are outside home? | 1. Yes  2. No |  |
| VI | Did your husband bath newborn/infant while you are busy? | 1. Yes  2. No |  |
| VII | Did your husband buy clothes/other things for infants/neonates? | 1. Yes  2. No |  |
| VIII | Did your husband go with you for immunization services? | 1. Yes  2. No |  |
| IX | Did your husband assisted you while you breastfeed the newborn? | 1. Yes  2. No |  |

**Part III: Decision making related questions**

| No | Questionnaire | Alternative choice for response | Skip code |
| --- | --- | --- | --- |
| 301 | Who decisions about health care for yourself? | 1. Me alone 2. My husband alone 3. Both of us 4. Others…………………………. |  |
| 302 | Who decides on large household purchase or sell? | 1. Me alone 2. My husband alone 3. Both of us 4. Others…………………………. |  |
| 303 | Who decides on intra household resource allocation/ daily household purchases? | 1. Me alone 2. My husband alone 3. Both of us 4. Others…………………………. |  |
| 304 | Who decides on where and when to seek medical care for sick newborns/children? | 1. Me alone 2. My husband alone 3. Both of us 4. Others…………………………. |  |
| 305 | Who decides on visits of family, friends or relatives? | 1. Me alone 2. My husband alone 3. Both of us 4. Others |  |
| 306 | Who decides when to have an additional child? | 1. Me alone 2. My husband alone 3. Both of us 4. Others…………………………. |  |
| 307 | Who usually decides how your partner’s/husband earnings will be used? | 1. Me alone 2. My husband alone 3. Both of us 4. Others…………………………. |  |
| 308 | Who decides to go for ANC visit, PNC visit, where to deliver and infant immunization? | 1. Me alone  2. My husband alone  3. Both of us  4. Others……………………………. |  |

| **Part IV: Social support scale Explanation of the Oslo-3 Social Support Scale** | | | | | | | |
| --- | --- | --- | --- | --- | --- | --- | --- |
| 401 | | How many people are so close to you that you can count on them if you have a serious problem? | 1. None  2. 1or 2  3. 3-5  4. 6 or more | | |  | |
| 402 | | How much concern do people show in what you are doing? | 1. No concern  2. Little concern and interest  3. Uncertain  4. Some concern and interest  5. A lot of concern and interest | | |  | |
| 403 | | How easy can you get practical help from neighbors if you should need it? | 1. Very difficult  2. Difficult  3. Possible  4. Easy  5. Very easy | | |  | |
| **Part V: Depression assessment tool (Patient health questionnaire-9)** | | | | 0 | 1 | | 2 |
| 501 | Little interest or pleasure in doing things | | |  |  | |  |
| 502 | Feeling down, depressed, or hopeless | | |  |  | |  |
| 503 | Staying asleep, or sleeping too much | | |  |  | |  |
| 504 | Feeling tired or having little energy | | |  |  | |  |
| 505 | Poor appetite or overeating | | |  |  | |  |
| 506 | Feeling bad about yourself, or have let yourself or your family down | | |  |  | |  |
| 507 | Trouble concentrating on things, such as reading the news paper  Or watching TV | | |  |  | |  |
| 508 | Moving or speaking so slowly that  Other people could have noticed. or the opposite being so fidgety or restless that you have been moving around a lot more than usual | | |  |  | |  |
| 509 | Thoughts that you would be better off dead, or of hurting yourself in some way | | |  |  | |  |

| **Part VI: About domestic/intimate partner violence** | | | |
| --- | --- | --- | --- |
| Psychological (emotional) violence: Has your partner ever….. | | | |
| 601 | Insulted/made feel bad about your-self? | 1.Yes  2. No |  |
| 602 | Belittled or humiliated in front of other people? | 1.Yes  2. No |  |
| 603 | Scared or intimidated on purpose? | 1.Yes  2. No |  |
| 604 | Threatened when asking friends/family? | 1.Yes  2. No |  |
| Physical violence: Has your partner ever….. | | | |
| 605 | Slapped you or thrown something at you that could hurt you? | 1.Yes  2. No |  |
| 606 | Pushed you or shoved or pulled your hair? | 1.Yes  2. No |  |
| 607 | Hit you with his fist or with something else that could hurt you? | 1.Yes  2. No |  |
| 608 | During pregnancy, hit your abdomen with a fist Or with something else? | 1.Yes  2. No |  |
| 609 | Choked or burnt you on purpose? | 1.Yes  2. No |  |
| 610 | Threatened to use or actually used a gun, knife, or any other weapon against you? | 1.Yes  2. No |  |
| Sexual violence: Has your partner ever….. | | | |
| 611 | Physically forced you to have sexual intercourse | 1.Yes  2. No |  |
| 612 | Having unwanted sexual intercourse because of fear from the partner | 1.Yes  2. No |  |
| 613 | Forced you to do something sexual that is degrading or humiliating | 1.Yes  2. No |  |
