# Supplementary material for: Low husband involvement in maternal and child health services and intimate partner violence increases the odds of postpartum depression in northwest Ethiopia: A community-based study
Source: PLoS One. 2022 Oct 26;17(10):e0276809. doi: 10.1371/journal.pone.0276809 (PMC9604988; doi:10.1371/journal.pone.0276809)
Supplement: Attachment — Submitted filename: PONE-D-21-35177_Reviewed.pdf [file pone.0276809.s003.pdf]

PLOS ONE

Low husband involvement in maternal and child health services and intimate partner violence increases the odds of postpartum depression in northwest Ethiopia: a community-based study

--Manuscript Draft--

Manuscript Number:	PONE-D-21-35177
Article Type:	Research Article
Full Title:	Low husband involvement in maternal and child health services and intimate partner violence increases the odds of postpartum depression in northwest Ethiopia: a community-based study
Short Title:	Postpartum depression and associated factors
Corresponding Author:	Nuhamin Tesfa Tsega, MPH<br>University of Gondar<br>Gondar, ETHIOPIA
Keywords:	Associated factor; Ethiopia; Depression; Husband involvement; Postpartum
Abstract:	Background<br><br>Depression is the most common mental health problem that affects women during pregnancy and after child-birth. Postpartum depression, in particular, has both short and long-term effects on the lives of mothers and children. Women’s health is a current global concern, but postpartum depression is a neglected issue in the maternal continuum of care and is rarely addressed. Therefore, this study aimed to assess postpartum depression and associated factors in Gondar city, northwest Ethiopia.<br><br>Methods<br><br>A community-based cross-sectional study was conducted from August 1st to 30th, 2021 in Gondar city. A cluster sampling technique was employed to select 794 postpartum women. Data were entered by EPI DATA version 4.6 and exported to SPSS version 25 for further analysis. The multivariable logistic regression analysis was carried out to identify factors associated with postpartum depression. The adjusted odds ratio with its 95% confidence interval at a p-value of ≤ 0.05 was used to declare e the level of significance.<br><br>Results<br><br>A total of 794 women were included in the analysis, giving a response rate of 98.5%. . The prevalence of postpartum depression was 17.25% (95% CI: 14.5, 20.2). Younger maternal age (AOR = 2.72, 95% CI: 1.23, 5.85), low average monthly income (AOR = 2.71, 95% CI: 1.24, 5.91), low decision-making power (AOR = 2.04, 95%CI: 1.31, 3.18), low husband/partner involvement in MNCH service (AOR = 2.34, 95%CI: 1.44, 3.81), unplanned pregnancy (AOR = 3.16 95% CI: 1.77, 5.62), and experience of intimate partner violence (AOR = 3.13; 95% CI: 1.96, 4.99) were significantly associated with postpartum depression.<br><br>Conclusion<br><br>In this study, nearly 1/5th of the study participants had postpartum depression. Thus, it is important to integrate maternal mental health services with the existing maternal health care services. It is also crucial to advocate the need for husband’s involvement in maternal and child health services and ensure women’s decision-making power in the household. Moreover, community-based sexual and reproductive health education would be better to reduce risk factors of postpartum depression.
Order of Authors:	Nuhamin Tesfa Tsega, MPH
	Azmeraw Ambachew Kebede, MSc
	Dereje Nibret Gessesse, MSc

Powered by Editorial Manager® and ProduXion Manager® from Aries Systems Corporation

	Mastewal Belayneh Aklil, MSc
	Wubedle Zelalem Temesgan, MSc
	Marta Yimam Abegaz, MSc
	Tazeb Alemu Anteneh, MSc
	Nebiyu Solomon Tibebu, MSc
	Haymanot Nigatu Alemu, MSc
	Tsion Tadesse Haile, MSc
	Asmra Tesfahun Seyoum, MSc
	Agumas Eskezia Tiguh, MSc
	Ayenew Engida Yismaw, MSc
	Muhabaw Shumye Mihret, MSc
	Goshu Nenko, MSc
	Kindu Yinges Wondie, MSc
	Birhan Tsegaw Taye, MSc
**Additional Information:**	
**Question**	**Response**
**Financial Disclosure**<br><br>Enter a financial disclosure statement that describes the sources of funding for the work included in this submission. Review the submission guidelines for detailed requirements. View published research articles from *PLOS ONE* for specific examples.<br><br>This statement is required for submission and **will appear in the published article** if the submission is accepted. Please make sure it is accurate.	The author(s) received no specific funding for this work.

**Unfunded studies**<br>Enter: *The author(s) received no specific funding for this work.*<br><br>**Funded studies**<br>Enter a statement with the following details:<br>• Initials of the authors who received each award<br>• Grant numbers awarded to each author<br>• The full name of each funder<br>• URL of each funder website<br>• Did the sponsors or funders play any role in the study design, data collection and analysis, decision to publish, or preparation of the manuscript?<br>• **NO** - Include this sentence at the end of your statement: *The funders had no role in study design, data collection and analysis, decision to publish, or preparation of the manuscript.*<br>• **YES** - Specify the role(s) played.<br><br>* typeset	
**Competing Interests**<br><br>Use the instructions below to enter a competing interest statement for this submission. On behalf of all authors, disclose any competing interests that could be perceived to bias this work—acknowledging all financial support and any other relevant financial or non-financial competing interests.<br><br>This statement is required for submission and **will appear in the published article** if the submission is accepted. Please make sure it is accurate and that any funding sources listed in your Funding Information later in the submission form are also declared in your Financial Disclosure statement.<br><br>View published research articles from *PLOS ONE* for specific examples.	The authors have declared that no competing interests exist.

Powered by Editorial Manager® and ProduXion Manager® from Aries Systems Corporation

**NO authors have competing interests**<br><br>Enter: *The authors have declared that no competing interests exist.*<br><br>**Authors with competing interests**<br><br>Enter competing interest details beginning with this statement:<br><br>*I have read the journal's policy and the authors of this manuscript have the following competing interests: [insert competing interests here]*<br><br>* typeset	
**Ethics Statement**<br><br>Enter an ethics statement for this submission. This statement is required if the study involved:<br><br>• Human participants<br>• Human specimens or tissue<br>• Vertebrate animals or cephalopods<br>• Vertebrate embryos or tissues<br>• Field research<br><br>Write "N/A" if the submission does not require an ethics statement.<br><br>General guidance is provided below. Consult the submission guidelines for detailed instructions. **Make sure that all information entered here is included in the Methods section of the manuscript.**	Ethical clearance was obtained from the University of Gondar Institutional Review Board (IRB) (Reference number: V/P/RCS/05/2710/2021). A formal letter of administrative approval was obtained from the selected clusters (kebeles) of Gondar city. Written informed consent was taken from each study participants after a clear explanation of the aim of the study.

Powered by Editorial Manager® and ProduXion Manager® from Aries Systems Corporation

**Format for specific study types**<br><br>**Human Subject Research (involving human participants and/or tissue)**<br>• Give the name of the institutional review board or ethics committee that approved the study<br>• Include the approval number and/or a statement indicating approval of this research<br>• Indicate the form of consent obtained (written/oral) or the reason that consent was not obtained (e.g. the data were analyzed anonymously)<br><br>**Animal Research (involving vertebrate animals, embryos or tissues)**<br>• Provide the name of the Institutional Animal Care and Use Committee (IACUC) or other relevant ethics board that reviewed the study protocol, and indicate whether they approved this research or granted a formal waiver of ethical approval<br>• Include an approval number if one was obtained<br>• If the study involved *non-human primates*, add *additional details* about animal welfare and steps taken to ameliorate suffering<br>• If anesthesia, euthanasia, or any kind of animal sacrifice is part of the study, include briefly which substances and/or methods were applied<br><br>**Field Research**<br><br>Include the following details if this study involves the collection of plant, animal, or other materials from a natural setting:<br>• Field permit number<br>• Name of the institution or relevant body that granted permission	
**Data Availability**<br><br>Authors are required to make all data underlying the findings described fully available, without restriction, and from the time of publication. PLOS allows rare exceptions to address legal and ethical concerns. See the PLOS Data Policy and FAQ for detailed information.	Yes - all data are fully available without restriction

A Data Availability Statement describing where the data can be found is required at submission. Your answers to this question constitute the Data Availability Statement and **will be published in the article**, if accepted.<br><br>**Important:** Stating 'data available on request from the author' is not sufficient. If your data are only available upon request, select 'No' for the first question and explain your exceptional situation in the text box.<br><br>Do the authors confirm that all data underlying the findings described in their manuscript are fully available without restriction?	
**Describe where the data may be found in full sentences. If you are copying our sample text, replace any instances of XXX with the appropriate details.**<br><br>• If the data are **held or will be held in a public repository**, include URLs, accession numbers or DOIs. If this information will only be available after acceptance, indicate this by ticking the box below. For example: *All XXX files are available from the XXX database (accession number(s) XXX, XXX.*).<br>• If the data are all contained **within the manuscript and/or Supporting Information files**, enter the following: *All relevant data are within the manuscript and its Supporting Information files.*<br>• If neither of these applies but you are able to provide **details of access elsewhere**, with or without limitations, please do so. For example:<br><br>*Data cannot be shared publicly because of [XXX]. Data are available from the XXX Institutional Data Access / Ethics Committee (contact via XXX) for researchers who meet the criteria for access to confidential data.*<br><br>*The data underlying the results presented in the study are available from (include the name of the third party*	All relevant data are within the manuscript and its Supporting Information files.

and contact information or URL).<br>• This text is appropriate if the data are owned by a third party and authors do not have permission to share the data.<br><br>* typeset	
Additional data availability information:	

manuscript Click here to access/download;Manuscript;Manuscript.docx

RESEARCH ARTCILE

Low husband involvement in maternal and child health services and intimate partner violence increases the odds of postpartum depression in northwest Ethiopia: a community-based study

Azmeraw Ambachew Kebede[1], Dereje Nibret Gessesse[1], Nuhamin Tesfa Tsega[2*], Mastewal Belayneh Aklil[1], Wubedle Zelalem Temesgan[1], Marta Yimam Abegaz[1], Tazeb Alemu Anteneh[1], Nebiyu Solomon Tibebu[1], Haymanot Nigatu Alemu[1], Tsion Tadesse Haile[1], Asmra Tesfahun Seyoum[1], Agumas Eskezia Tiguh[1], Ayenew Engida Yismaw[1], Muhabaw Shumye Mihret[1], Goshu Nenko[3], Kindu Yinges Wondie[1], Birhan Tsegaw Taye[4]

[1] Department of Clinical Midwifery, School of Midwifery, College of Medicine and Health Sciences, University of Gondar, Gondar, Ethiopia; [2] Department of Women's and Family Health, School of Midwifery, College of Medicine and Health Sciences, University of Gondar, Gondar, Ethiopia; [3] Department of Psychiatry, College of Medicine and Health Sciences, University of Gondar, Gondar, Ethiopia; [4] Department of Midwifery, College of Health Sciences, Debre Berhan University, Debre Berhan, Ethiopia

[*] nuha26tesfa@gmail.com

Abstract

Background

Depression is the most common mental health problem that affects women during pregnancy and after child-birth. Postpartum depression, in particular, has both short and long-term effects on the lives of mothers and children. Women's health is a current global concern, but postpartum depression is a neglected issue in the maternal continuum of care and is rarely addressed. Therefore, this study aimed to assess postpartum depression and associated factors in Gondar city, northwest Ethiopia.

Methods

A community-based cross-sectional study was conducted from August 1st to 30th, 2021 in Gondar city. A cluster sampling technique was employed to select 794 postpartum women. Data were entered by EPI DATA version 4.6 and exported to SPSS version 25 for further analysis. The multivariable logistic regression analysis was carried out to identify factors associated with postpartum depression. The adjusted odds ratio with its 95 % confidence interval at a p-value of ≤ 0.05 was used to declare e the level of significance.

Results

A total of 794 women were included in the analysis, giving a response rate of 98.5%. . The prevalence of postpartum depression was 17.25% (95% CI: 14.5, 20.2). Younger maternal age (AOR = 2.72, 95% CI: 1.23, 5.85), low average monthly income (AOR = 2.71, 95% CI: 1.24, 5.91), low decision-making power (AOR = 2.04, 95%CI: 1.31, 3.18), low husband/partner involvement in MNCH service (AOR = 2.34, 95%CI: 1.44, 3.81), unplanned pregnancy (AOR = 3.16 95% CI: 1.77, 5.62), and experience of intimate partner violence (AOR = 3.13; 95% CI: 1.96, 4.99) were significantly associated with postpartum depression.

Conclusion

In this study, nearly 1/5th of the study participants had postpartum depression. Thus, it is important to integrate maternal mental health services with the existing maternal health care services. It is also crucial to advocate the need for husband's involvement in maternal and child

health services and ensure women's decision-making power in the household. Moreover, community-based sexual and reproductive health education would be better to reduce risk factors of postpartum depression.

Key words: Associated factor, Ethiopia, Depression, Husband involvement, Postpartum

Introduction

Depression is the most common psychiatric condition that occurs during pregnancy and the postpartum period (1). Postpartum Depression (PPD) is defined as depressive symptoms such as reduced mood, loss of enjoyment, diminished energy and activity, functional impairment, low self-esteem, and suicidal thoughts or acts that occur within the first year after childbirth (2-4). The transition to motherhood is considered to be a difficult and emotional transition with significant changes in psychological, social and physiological aspects, and increased susceptibility to mental illnesses including PPD (5). It is a major public health concern because it affects mothers and newborns, as well as family members and the society at large (6). In the same way, intimate partner violence (IPV) is a global public health problem within an intimate relationship that causes physical, psychological, or sexual harm (7). It can cause extensive mental health consequences including depression among victims (8, 9).

Around 10%-20% of mothers suffer from depressive symptoms after childbirth worldwide (10). In sub-Saharan Africa the magnitude of postpartum depression is 18.6% (11). A meta-analysis study conducted in Ethiopia also showed that 21.5% of women develop postpartum depression (12).

Empirical evidence has found that postpartum depression is linked to impaired mother-infant bonding, child abuse, child neglect, maternal substance abuse, and self-harm (13, 14). In addition, maternal depression has also been linked to poor weight gain, impaired cognitive and motor development in infants, and early discontinuation of breastfeeding because of reduced breast milk production (13, 15). Moreover, maternal depression affects the nutrition of the women that could lead to some morbid conditions like anemia, malnutrition, and hypertension (1, 10). The family can be affected through neglect of family duties and financial strain due to the treatment costs for PPD and low productivity at work (10). This maternal depression will also lead to suicide, which is a leading cause of death in the first postnatal year, accounting for

It can lead to suicide, but not in all or even most cases, so you shouldn't say it will.

around 22% of maternal deaths. About 10% of maternal suicide, in particular, is resulted from postpartum mental health problems (16).

Endless crying of babies, painful and cracked nipples, painful delivery wounds, inadequate breast milk, family demands, sleepless nights, and constant fatigue have been associated with postpartum depression (17). Even though PPD has no single cause, some of the factors are being a first-time mother, history of previous depression (18), domestic violence (19, 20), history of substance use, poor social support (20, 21), and unplanned pregnancy (18, 19).

The 2015 World Health Organization recommendation on measures to promote maternal and child health outcomes during pregnancy, childbirth and the postpartum period include effective

Male involvement? Sounds clearer

implementation of men involvement in the maternal continuum of care (22). This is because low husband/partner involvement in maternal and child health care services during pregnancy and the postpartum period is a leading cause of poor maternal health, including PPD (23).

Do you mean poor maternal health in general or only poor maternal mental health here?

However, the PPD screening tool is not incorporated into modern postnatal care guidelines in

Which PPD screening tool? I am aware of more than one, so please specify which you are talking about here.

Ethiopia. This study can help reduce maternal PPD and influencing factors and provide information on strategies targeting on maternal and child health. Therefore, this community based cross-sectional study assessed postnatal depression and associated factors among women who gave birth in the last one year in Gondar City, Northwest Ethiopia.

Method and materials

Study design and period

A community-based cross-sectional study was conducted in Gondar city from August 1st to August 30th, 2021.

Study area

Gondar city is found in Amhara national regional state, Central Gondar Zone. It is located 166 km from Bahir Dar (the capital city of Amhara regional state) and 750 km Northwest of Addis Ababa (the capital city of Ethiopia). According to the population projection of Ethiopia, the estimated total population of the city was 432,191, of whom, 224,508 are females. From this, about 133, 477 (30.88%) of females are in the reproductive age (unpublished data by Amhara

How are you defining reproductive age?

regional state, 2021). There are 1 governmental comprehensive specialized referral hospital, 8 governmental health centers, 22 health posts, 1 private primary hospital, and 1 general hospital serving the town.

Study population and eligibility criteria

Put actual dates

The study population included all women who gave birth in the last year and who resided in the city for at least 6 months in the selected kebeles during the data collection period.

Sample size determination and sampling procedure

The sample size was determined by using the single population proportion formula by considering the following assumptions: the proportion of postpartum depression 33.82% (24),

Where did this specific number come from?

level of confidence 95%, and margin of error 5%. Therefore, the sample size (n) = $\frac{(Z\alpha/2)^2 p(1-p)}{d^2} = \frac{(1.96)^{2*}0.3382(1-0.3382)}{(0.05)2} = 344$. After considering a design effect of 2 and a non-response rate of 10 %, the total sample size was 757. Gondar city has 22 kebeles and six kebeles (25% of the total kebeles) were selected by a lottery method. All eligible women in the selected clusters were interviewed. Finally, due to the nature of cluster sampling, 806 women were included in our study.

Variables of the study

Postpartum depression was the outcome variable whereas maternal age, religion, marital status, mother's educational status, women's occupation, average monthly income, mother's educational status, husband educational status, husband occupation, parity, having ANC visit,

Did you only look at one ANC visit? Or the recommended number?

place of delivery, mode of delivery, PNC visit, planned pregnancy, intimate partner violence (IPV), decision-making power, social support, and husband/partner involvement in MNCH, family history of mental health problem, known psychiatric illness, having information about mental health during pregnancy, medical disorder and experienced a death of family or friends were independent variable of the study.

Operational definitions and measurements

Postpartum depression: women who were interviewed and scored ten and above by using patient health questionnaire-9 (PHQ-9) were considered as depressed (25).

Social support: The Oslo Social Support Scale (OSS-3) scores ranged from 3-14 with a score of 3-8, poor support; 9-11, moderate support; and 12-14, strong support (26).

This is confusingly worded.

Household decision-making power: The ability of women to act independently on the household activities including their health, children's health, freedom of movement, and control over finance, decides on large household purchases. Thus, based on the summative score of variables designed to assess household decision-making power women who were answered above the mean value were considered as having higher decision-making power (27).

What was that value? What was the range? I am confused about the scores on this.

Husband/partner involved in MNCH services: Based on the summative score of variables designed to assess husband involvement with a score above the mean was considered as involved (23).

See above

Intimate partner violence: Intimate partner is considered as a current spouse, co-habited, current boyfriends, former partner, or spouse. Women were considered to have experienced intimate partner violence, if they said "Yes" to any one of the ranges of sexual, psychological, and physical or any combination of the three coercive acts regardless of the legal status of the relationship with current/former intimate partner, it was considered as IPV (28).

Data collection tool and quality assurance

The data collection tool was developed by reviewing the literature (8, 19-21, 24, 29-33) and was collected using a structured questionnaire through face-to-face interviews. The questionnaire was prepared in the English version and translated to the local language (Amharic) and back to English to keep uniformity. The questionnaire contains socio-demographic characteristics, obstetric, medical, and maternal health services-related characteristics, social support, husband/partner involvement in MNCH, decision-making power, and intimate partner violence-related questions, and questions assessing PPD. Six BSc and 2 MSc midwives were recruited for data collection and supervision, respectively. One day training was given for data collectors and supervisors about the interview technique and supervising the data process. The pretest was done

Who were the interviewers?

on 5% of the determined sample size in the Maksegnit district to look for the understandability and appropriateness of the study tool. The completeness of the questionnaire was checked by the supervisors daily.

Data management and analysis

Data were checked, coded, and entered into EPI Data version 4.6 and exported to SPSS version 25 for further analysis. Descriptive statistics like frequency, mean, and proportion were used to present participants' characteristics. Binary logistic regression was fitted to identify eligible factors and variables having a p-value of ≤ 0.2 were included in the multivariable logistic regression analysis. In the multivariable logistic regression analysis, a p-value of ≤ 0.05 with a 95% CI for the adjusted odds ratio was used to claim the level of significance.

Ethical considerations

Ethical clearance was obtained from the University of Gondar Institutional Review Board (IRB) (Reference number: V/P/RCS/05/2710/2021). A formal letter of administrative approval was obtained from the selected clusters (kebeles) of Gondar city. Written informed consent was taken from each study participants after a clear explanation of the aim of the study.

Result

Socio demographic characteristics of respondents

A total of 806 women were included in the study making a response rate of 98.5%. Among the total study participants, the mean age of the respondents was 29.7 years old (±SD 4.83) and 550 (69.3%) of the respondents were in the age group of 25-34 years. Most (81.6%) of the study participants were Orthodox Christian. Regarding marital status, 718 (90.4%) of study participants were married. More than one quarter, 229 (28.8%) of study participants had completed secondary education. Regarding occupation, 357 (45%) women were housewives, and 457(66.15%) of their husbands were government employees **(Table 1)**.

Obstetrics, medical related, and maternal health service characteristics

Among the total respondents, more than half (56.2%) of women had a parity of two to four. The majority, 773 (97.4%) of study participants had at least one ANC follow up whereas 418 (52.6%)
the use of the term ANC follow up is confusing as a follow up is usually an appointment after a medical event, and ANC is before the birth.
of the participants had at least one PNC visit. Six hundred seventy eight (85.4%) and seven hundred thirty seven (92.8%) of the pregnancy was planned and supported by husband/family,
pregnancies were
respectively. Regarding social support, 228 (28.7%) of the respondents had poor social support. More than half, 416 (52.4%) of women's got their husband/partner's support during maternal, neonatal, and child health care services, and nearly two-thirds, 494 (62.2%) of women had a higher decision making power **(Table 2)**.

Magnitude of postpartum depression and associated factors

Prevalence?
The magnitude of postpartum depression among women who gave birth in the last one year was 17.25% (95% CI: 14.5, 20.2). In the binary logistic regression analysis: maternal age, women's educational status, family monthly income, intimate partner violence, women's decision-making power, type of pregnancy, husband/partner involvement in MNCH service, social support, place of delivery, and pregnancy supported by family/partner were found to be a p-value of <0.2 and entered to multivariable analysis. However, maternal age, family monthly income, women's decision-making power, husband/partner involvement in MNCH care service, type of pregnancy, and IPV were significantly associated with postpartum depression in the multivariable analysis.

Women whose age was ≤ 24 years were 2.72 times more likely to develop postpartum depression compared with women whose age was ≥ 35 years (AOR = 2.72, 95% CI: 1.23, 5.85). Women whose average monthly income ≤ 1000 Ethiopian Birr (ETB) were 2.71 times more likely to experience postpartum depression than those women who earned > 2000 ETB (AOR = 2.71, 95% CI: 1.24, 5.91). This study revealed that women who had lower decision-making power were 2.04 times more likely to have had depression during the postpartum period compared with those women who had higher decision-making power (AOR = 2.04, 95% CI: 1.31, 3.18).

Women whose husband/partner were not actively involved in MNCH service were 2.34 times more likely to experience postpartum depression compared with those women whose husbands/partners were actively involved in MNCH care services (AOR = 2.34, 95%CI: 1.44,

3.81). The current study revealed that type of pregnancy has been strongly associated with postpartum depression. The odds of experiencing postpartum depression among respondents who had unplanned pregnancies were 3 times higher as compared to those women who had planned pregnancies (AOR = 3.16 95% CI: 1.77, 5.62). The study also declared that there was a significant association between IPV and postpartum depression. Thus, the odds of having postpartum depression among women who had IPV was about 3 times higher compared with women who didn't have IPV (AOR = 3.13; 95% CI: 1.96, 4.99) **(Table 3).**

Found? a study can't really declare something

Discussion

A large portion of this section focuses on comparing the prevalence of PPD in this study to other studies. Based on the beginning of this paper, determining the prevalence of PPD isn't the main purpose of the study, so talking about that for so long isn't adding anything to the discussion. You aren't talking about what this study is actually adding to the literature. What new information did we get from this study? And what can we do with that information?

This community-based cross-sectional study assessed postpartum depression and associated factors among women who gave birth in the last one year in Gondar city, northwest Ethiopia, 2021. Thus, it was found that the prevalence of postpartum depression was 17.25% (95% CI: 14.5, 20.2), which is comparable with studies conducted in Debre Berhan, Ethiopia-15.6% (34) and Eastern Ethiopia-16.3% (35).

Again, use specific dates

However, the finding of this study was higher than studies conducted in Hiwot Fana Specialized Hospital, Ethiopia-13.11% (36), Eritrea-7.4% (37), Kenya-13.0% (31), and South Africa-8.8% (38). The possible reason for this discrepancy could be due to study setting and socio-cultural differences. All the above-mentioned studies were institution-based cross-sectional studies where women who came for MNCH care services will get health education about the physiologic and psychologic changes during the postpartum period. In addition, IPV has been linked with mental health problems, particularly postpartum depression (39). For instance, nearly half (48.9%) of the study participants in the current study have experienced IPV, while only 3.7% of women in South Africa experienced IPV.

I assume you are still comparing PPD prevalence at this point? This study had many findings, so be specific

On the other hand, the result of this study was lower compared with other studies conducted somewhere else in Ethiopia including Gondar town-25% (40), Awi Zone-23.7% (41), Bench Maji Zone-22.4% (21), and Southwest Ethiopia-33.8% (24). The result of this study is also lower as compared to a study conducted in Cameron-23.4% (32). This variation might be due to the differences in the tool we used to measure the outcome variable, time of data collection, and characteristics of the study participants. The study conducted in Awi Zone, Ethiopia used Edinburgh Postnatal Depression Scale (EPDS) with a cutoff point of 8 to declare postpartum depression. In this study, however, the PHQ-9 depression scale with a cutoff point of 10 was

Again, see above about specificity

used. Moreover, the study participants in Gondar town and Cameron were women who gave birth in the last 6 weeks and women whose children aged 4 to 6 weeks respectively. Because postpartum depression is most common in the first 6 weeks after child birth (21). On the other hand, the study in Bench Maji Zone, Ethiopia showed that 42.1 % of the study participants were under the age of 23 years. However, only 11.2% of the participants were under the age of 24 in this study. It has been evidenced that being younger age is highly correlated with postpartum depression (29, 42). The lower prevalence of PPD in the current study might also be related to the low incidence of unplanned pregnancy. Unplanned and unsupported pregnancies have been associated with PPD as evidenced by scholars (20, 21, 43, 44). Hence, 30% of the pregnancy in the Southwest Ethiopia study was unplanned whereas only 14.6% of the pregnancies were unplanned in our study.

incomplete sentence

It has been found that being younger age increases the odds of developing PPD. Accordingly, the odds of having postpartum depression was 2.72 times higher among women aged ≤ 24 years compared with those women aged ≥ 35 years old. This finding was supported by the study conducted in Southwest Ethiopia (21), Sudan (30), and Armenia (29). The possible explanation might be due to the fact that younger women are expected to be exposed to emotional distress as they experience childbirth for the first time. Besides, the additional burden of caring for infants and preparation to be a mother will be often challenging and will lead to unpleasant health outcomes (45). In this regard, the need to screen younger women for mental health problems and endorsing screening tools starting from the prenatal period will be crucial.

The study also affirmed that the family monthly income of the respondents was one of the variables positively associated with PPD. Respondents who had an average monthly income ≤ 1000 ETB were 2.7 times more likely to report PPD than participants who had an average monthly income of >2000 ETB. This finding was supported by studies conducted in Kenya (31) and Cameron (32). This might be due to women with low socioeconomic status may face difficulties to fulfill their needs and newborns during the postpartum period. Low socioeconomic status has been connected with a high rate of different mental health problems (46, 47).

This study also revealed that women who had lower decision-making power were 2.04 times more likely to have had depression during the postpartum period compared with those women who had higher decision-making power. This result is consistent with studies done in Bahir Dar,

Ethiopia (19), and China (33). The possible reason for this could be those women who didn't have power and control over resources, restriction to access maternal and reproductive health services, and unable to decide independently for their health could negatively affect their overall wellbeing. This could also be justifies as about 45% of the participants in this study were housewives, in which being unemployed has been associated with mental health problems so far (48). This is because employed women are expected to have higher levels of household decision-making power as compared with their counterparts. Unemployed women are usually economically dependent on their husband/partner, particularly in developing countries and exclude women from different opportunities (49).

In this study, husband/partner involvement in MNCH service was significantly associated with PPD. Accordingly, women whose husbands/partners were not actively involved in MNCH care service were 2.34 times more likely to experience postpartum depression compared with those women whose husbands/partners were involved in MNCH care service. This could be explained by being supported by a husband or partner will build a higher sense of support to the women.

This sentence is confusingly worded

Evidence support that husband/partner involvement in MNCH services is found to be crucial for the reduction of adverse health outcomes (50). This is because men are influential in making health care decisions that may make the women feeling of under estimated often leading to

Also confusingly wprded

mental health problems. However, only 52.4% of husbands/partners have been involved in MNCH care service in the current study.

The current study revealed that the type of pregnancy has been strongly associated with PPD. Thus, the odds of experiencing PPD among respondents who had unplanned pregnancy was 3 times more compared with those women who had planned pregnancy. This result is supported by studies conducted in Ethiopia such as Bahir Dar (19), Nekemte town (20), Bench Maji zone, (21), and southwest Ethiopia (24), and in Pennsylvania (51). This could be due to the fact that pregnancy itself needs physiological, psychological, and financial preparation. Empirical evidence showed that unplanned pregnancy is associated with PPD (43, 44).

This study also declared that there was a significant association between IPV and PPD. Hence, the odds of having PPD among women who had IPV was 3 times higher compared with those women who didn't have intimate partner violence. Similar findings were reported from previous studies done in Nekemte town (20), Bahir Dar town, Ethiopia (19), and Bangladesh (8). The

explanation for this might be IPV has a major effect on women’s physical and psychological health and this may lead to postpartum depression (9). This indicates that screening for IPV in antenatal and postnatal care could help to identify and treat women at risk of depression. Lastly, the authors would like to acknowledge the limitation of this study. Due to the cross-sectional nature of the study, it couldn’t be possible to infer cause-effect between the outcome and explanatory variables. There are additional study limitations. For example, the use of interviewer-delivered surveys can lead to issues like social desirability bias or interviewer bias.

Conclusion

What additional research is needed? Are there any specific activities you would recommend? You mentioned that a screening tool for PPD wasn’t included in the guidelines for care in Ethiopia. Would you recommend it is? If so, how do you recommend that be done? I am left at the end of this study think “so what” because it isn’t clear what exactly you want done with this information or how it can be built on.

In this study, PPD was comparable with previous studies. However, given the community health context, the magnitude is even higher. Being younger, lack of husband involvement in MNCH care services, lack of decision-making power in the household, experiencing IPV, unplanned pregnancy, and lower household monthly income were found to increase the odds of experiencing PDD. It is important to integrate mental health with maternal and child health care service guidelines for screening, timely transfer, and early treatment of those women who are at risk of postpartum depression. It is also crucial to advocate the need for the husband’s/partner‘s involvement in maternal and child health services and ensure women’s decision-making power in the household. Moreover, community-based sexual and reproductive health education would be better to reduce risk factors for postpartum depression.

Supporting information

S1 file: English version of the questionnaire.

S2 file: SPSS dataset.

Acknowledgments

We would like to thank the University of Gondar for providing study ethical clearance to conduct this study. Our gratitude also goes to all data collectors and study participants. We are glad to Gondar city kebeles for writing permission letter.

Authors’ Contributions

Conceptualization: Azmeraw Ambachew Kebede

Data curation: Azmeraw Ambachew Kebede, Dereje Nibret Gessesse, Nuhamin Tesfa Tsega, Mastewal Belayneh Aklil, Wubedle Zelalem Temesgan, Marta Yimam Abegaz, Tazeb Alemu Anteneh, Nebiyu Solomon Tibebu, Haymanot Nigatu Alemu, Tsion Tadesse Haile, Asmra Tesfahun Seyoum, Agumas Eskezia Tiguh, Ayenew Engida Yismaw, Muhabaw Shumye Mihret, Goshu Nenko, Kindu Yinges Wondie, Birhan Tsegaw Taye

Formal analysis: Azmeraw Ambachew Kebede, Dereje Nibret Gessesse, Nuhamin Tesfa Tsega, Mastewal Belayneh Aklil, Wubedle Zelalem Temesgan, Marta Yimam Abegaz, Tazeb Alemu Anteneh, Nebiyu Solomon Tibebu, Haymanot Nigatu Alemu, Tsion Tadesse Haile, Asmra Tesfahun Seyoum, Agumas Eskezia Tiguh, Ayenew Engida Yismaw, Muhabaw Shumye Mihret, Goshu Nenko, Kindu Yinges Wondie, Birhan Tsegaw Taye

Investigation: Azmeraw Ambachew Kebede, Dereje Nibret Gessesse, Nuhamin Tesfa Tsega, Mastewal Belayneh Aklil, Wubedle Zelalem Temesgan, Marta Yimam Abegaz, Tazeb Alemu Anteneh, Nebiyu Solomon Tibebu, Haymanot Nigatu Alemu, Tsion Tadesse Haile, Asmra Tesfahun Seyoum, Agumas Eskezia Tiguh, Ayenew Engida Yismaw, Muhabaw Shumye Mihret, Goshu Nenko, Kindu Yinges Wondie, Birhan Tsegaw Taye

Methodology: Azmeraw Ambachew Kebede, Dereje Nibret Gessesse, Nuhamin Tesfa Tsega, Mastewal Belayneh Aklil, Wubedle Zelalem Temesgan, Marta Yimam Abegaz, Tazeb Alemu Anteneh, Nebiyu Solomon Tibebu, Haymanot Nigatu Alemu, Tsion Tadesse Haile, Asmra Tesfahun Seyoum, Agumas Eskezia Tiguh, Ayenew Engida Yismaw, Muhabaw Shumye Mihret, Goshu Nenko, Kindu Yinges Wondie, Birhan Tsegaw Taye

Validation: Azmeraw Ambachew Kebede, Dereje Nibret Gessesse, Nuhamin Tesfa Tsega, Mastewal Belayneh Aklil, Wubedle Zelalem Temesgan, Marta Yimam Abegaz, Tazeb Alemu Anteneh, Nebiyu Solomon Tibebu, Haymanot Nigatu Alemu, Tsion Tadesse Haile, Asmra Tesfahun Seyoum, Agumas Eskezia Tiguh, Ayenew Engida Yismaw, Muhabaw Shumye Mihret, Goshu Nenko, Kindu Yinges Wondie, Birhan Tsegaw Taye

Visualization: Azmeraw Ambachew Kebede, Dereje Nibret Gessesse, Nuhamin Tesfa Tsega, Mastewal Belayneh Aklil, Wubedle Zelalem Temesgan, Marta Yimam Abegaz, Tazeb Alemu Anteneh, Nebiyu Solomon Tibebu, Haymanot Nigatu Alemu, Tsion Tadesse Haile, Asmra Tesfahun Seyoum, Agumas Eskezia Tiguh, Ayenew Engida Yismaw, Muhabaw Shumye Mihret, Goshu Nenko, Kindu Yinges Wondie, Birhan Tsegaw Taye

Writing-original draft: Nuhamin Tesfa Tsega, Wubedle Zelalem Temesgan, Tazeb Alemu Anteneh, Azmeraw Ambachew Kebede

Writing- review and editing: Azmeraw Ambachew Kebede, Dereje Nibret Gessesse, Nuhamin Tesfa Tsega, Mastewal Belayneh Aklil, Wubedle Zelalem Temesgan, Marta Yimam Abegaz, Tazeb Alemu Anteneh, Nebiyu Solomon Tibebu, Haymanot Nigatu Alemu, Tsion Tadesse Haile, Asmra Tesfahun Seyoum, Agumas Eskezia Tiguh, Ayenew Engida Yismaw, Muhabaw Shumye Mihret, Goshu Nenko, Kindu Yinges Wondie, Birhan Tsegaw Taye

Data Availability Statement: All relevant data are within the manuscript and its Supporting information files.

Funding: The authors received no specific funding for this work.

Competing interests: The authors declare that they have no conflict of interest in this work.

Abbreviations

AOR: Adjusted Odds Ratio; ANC: Antenatal Care; CI: Confidence Interval; COR: Crude Odds Ratio; IPV: Intimate Partner Violence; MNCH: Maternal, Neonatal, and Child Health; PNC: Postnatal Care; PPD: Postpartum Depression; SPSS: Statistical Package for Social Science;

Table 1: Socio-demographic characteristics of women who gave birth in the last one year in Gondar city, Northwest Ethiopia, 2021 (n = 794)

Variables	Frequency	Percentage (%)
**Age**		
≤24	98	12.3
25-34	550	69.3
≥35	146	18.4
**Religion**		
Orthodox Christian	648	81.6
Muslim<br>Protestant	105<br>32	13.2<br>4.0
Others[a]	9	1.1
**Current marital status**		
Married	718	90.4
Unmarried	76	9.6
**Women's education status**		
No formal education	96	12.1
Primary education	128	16.1
Secondary education	229	28.8
Diploma and above	341	42.9
**Women's occupation**		
Housewives	357	45
Daily laborer	14	1.8
Self-employee	97	12.2
Merchant	97	12.2
Government employee	215	27.1
**Husband educational status (n = 718)**		
No formal education	40	5.57
Primary education	54	7.52
Secondary education	149	20.75

Diploma and above	457	66.15
**Husband occupation (n = 718)**<br>Daily labor	 45	 6.27
Self-employee	161	22.42
Government employee	333	46.37
Merchant	151	21.03
Others[b]	28	3.89
**Average family monthly income**		
≤1000 ETB	49	6.2
1001-2000 ETB	53	6.7
≥2001 ETB	692	87.2

ETB: Ethiopian Birr, a: Jewish and Adventist, b: Student and Farmer

Table 2: Obstetrics, medical related and maternal health service characteristics of women who gave birth in the last one year in Gondar city, northwest Ethiopia, 2021 (n = 794)

**Variables**	**Frequency**	**Percentage (%)**
**Parity**		
1	318	40.1
2-4	446	56.2
≥5	30	3.8
**ANC follow up** Again, use of the term follow up when talking about ANC is not appropriate		
Yes	773	97.4
No	21	2.6
**Place of delivery**		
Home	24	3
Health facility	770	97
**Mode of delivery**		
SVD	471	59.3
Cesarean section	297	37.4
Instrumental delivery	26	3.3
**PNC visit**		

Yes	418	52.6
No	376	47.4
**Type of pregnancy**		
Planned	678	85.4
Unplanned	116	14.6
**Was the pregnancy supported**		
Yes	737	92.8
No	57	7.2
**Women's decision making power**		
Higher	494	62.2
Lower	300	37.8
**Husband/partner involvement in MNCH**		
Involved	416	52.4
Not involved	378	47.6
**Social support**		
Poor	228	28.7
Moderate	351	44.2
Strong	215	27.1
**Intimate partner violence**		
Yes	388	48.9
No	406	51.1
**Family history of mental health problem**		
Yes	83	10.5
No	711	89.5
**Known psychiatric illness**		
Yes	19	2.4
No	775	97.6
**Having information about mental health during pregnancy**		
Yes	365	46
No	429	54

Known medical disorder How were you defining medical disorder? That could mean many different things.		
Yes	86	10.8
No	708	89.2
**Experienced death of husband/friend/relatives**		
Yes	202	25.4
No	592	74.6

ANC: Antenatal Care, SVD: Spontaneous Vaginal Delivery, PNC: Postnatal Care, MNCH: Maternal, Neonatal and Child Health

Table 3: Bivariable and multivariable logistic regression analysis of associated factors of postpartum depression among women who gave birth in the last 1 year in Gondar city, northwest Ethiopia, 2021 (n = 794)

Variables	**Postpartum depression**		COR (95%CI)	AOR (95% CI)
	**Yes**	**No**		
**Material age**				
≤ 24	31	67	3.09 (1.63, 5.88)	2.72 (1.23, 5.85)*
25-34	87	463	1.26 (0.74, 2.14)	1.35 (0.73, 2.51)
≥ 35	19	127	1	1
**Women's education status**				
No formal education	30	66	3.97 (2.28, 6.93)	1.16 (0.57, 2.39)
Primary education	18	110	1.43 (0.78, 2.63)	0.49 (0.24, 1.02)
Secondary education	54	175	2.69 (1.69, 4.29)	1.21 (0.71, 2.08)
Diploma and above	35	306	1	1
**Family monthly income**				
≤ 1000 ETB	21	28	4.24 (2.32, 7.75)	2.71 (1.24, 5.91)*
1001-2000 ETB	12	41	1.66 (0.84, 3.25)	0.92 (0.42, 2.02)
≥ 2001 ETB	104	588	1	1
**Place of delivery**				
Home	9	15	3.01 (1.29, 7.03)	2.36 (0.89, 6,29)
Health facility	128	642	1	1
**Social support**				

Poor	64	164	2.97 (1.79, 4.93)	1.53 (0.84, 2.77)
Moderate	48	303	1.20 (0.72, 2.02)	1.11 (0.62, 1.98)
Strong	25	190	1	1
**Women's decision-making power**				
Higher	61	433	1	1
Lower	76	224	2.41 (1.66, 3.49)	2.04 (1.31, 3.18)*
**Husband/partner involvement in MNCH**				
Involved	43	373	1	1
Not involved	94	284	2.87 (1.94, 4.25)	2.34 (1.44, 3.81)**
**Type pregnancy**				
Planned	86	592	1	1
Unplanned	51	65	5.40 (3.51, 8.31)	3.16 (1.77, 5.62)**
**Was the recent pregnancy supported**				
Yes	118	619	1	1
No	19	38	2.62 (1.46, 2.71)	0.53 (0.23, 1.19)
**Intimate partner violence**				
Yes	104	284	4.41 (2.71, 6.31)	3.13 (1.96, 4.99)**
No	33	373	1	1

NB: * Significant (P-value ≤ 0.05), ** P-value <0.001

MNCH: Maternal, Neonatal, and Child Health, AOR: Adjusted Odd Ratio, COR: Crude Odd Ratio, CI: Confidence Interval

References

1. Parsons CE, Young KS, Rochat TJ, Kringelbach ML, Stein A. Postnatal depression and its effects on child development: a review of evidence from low-and middle-income countries. British medical bulletin. 2012;101(1):57-79.
2. Turkcapar AF, Kadıoğlu N, Aslan E, Tunc S, Zayıfoğlu M, Mollamahmutoğlu L. Sociodemographic and clinical features of postpartum depression among Turkish women: a prospective study. BMC Pregnancy & Childbirth. 2015;15(1):1-8.
3. Lund C, Town C. Maternal depression. Health And Education Advice & Resource Team. 2016:1-7.
4. Guo N, Bindt C, Te Bonle M, Appiah-Poku J, Hinz R, Barthel D, et al. Association of antepartum and postpartum depression in Ghanaian and Ivorian women with febrile illness in their offspring: a prospective birth cohort study. American journal of epidemiology. 2013;178(9):1394-402.
5. Chowdhary N, Sikander S, Atif N, Singh N, Ahmad I, Fuhr DC, et al. The content and delivery of psychological interventions for perinatal depression by non-specialist health workers in low and middle income countries: a systematic review. Best practice & research Clinical obstetrics & gynaecology. 2014;28(1):113-33.
6. Connelly CD, Hazen AL, Baker-Ericzén MJ, Landsverk J, Horwitz SM. Is screening for depression in the perinatal period enough? The co-occurrence of depression, substance abuse, and intimate partner violence in culturally diverse pregnant women. Journal of Women's Health. 2013;22(10):844-52.
7. WHO. Understanding and addressing violence against women: 2012 [Available from: https://apps.who.int/iris/bitstream/handle/10665/77432/WHO_RHR_12.36_eng.pdf.
8. Azad R, Fahmi R, Shrestha S, Joshi H, Hasan M, Khan ANS, et al. Prevalence and risk factors of postpartum depression within one year after birth in urban slums of Dhaka, Bangladesh. PloS one. 2019;14(5):e0215735.
9. Ziaei S, Frith AL, Ekström E-C, Naved RT. Experiencing lifetime domestic violence: associations with mental health and stress among pregnant women in rural Bangladesh: the MINIMat Randomized Trial. PLoS one. 2016;11(12):e0168103.
10. Azale T, Fekadu A, Hanlon C. Treatment gap and help-seeking for postpartum depression in a rural African setting. BMC psychiatry. 2016;16(1):1-10.

11. Woldeyohannes D, Tekalegn Y, Sahiledengle B, Ermias D, Ejajo T, Mwanri L. Effect of postpartum depression on exclusive breast-feeding practices in sub-Saharan Africa countries: a systematic review and meta-analysis. BMC Pregnancy and Childbirth. 2021;21(1):1-10.
12. Desta M, Memiah P, Kassie B, Ketema DB, Amha H, Getaneh T, et al. Postpartum depression and its association with intimate partner violence and inadequate social support in Ethiopia: a systematic review and meta-analysis. Journal of affective disorders. 2020.
13. Eastwood J, Ogbo FA, Hendry A, Noble J, Page A, Group EYR. The impact of antenatal depression on perinatal outcomes in Australian women. PLoS One. 2017;12(1):e0169907.
14. Desmarais SL, Pritchard A, Lowder EM, Janssen PA. Intimate partner abuse before and during pregnancy as risk factors for postpartum mental health problems. BMC pregnancy and childbirth. 2014;14(1):1-12.
15. Perfetti J, Clark R, Fillmore C-M. Postpartum depression: identification, screening, and treatment. WMJ-MADISON-. 2004;103:56-63.
16. Knight M, Kenyon S, Brocklehurst P, Neilson J, Shakespeare J, Kurinczuk JJ. Saving lives, improving mothers' care-lessons learned to inform future maternity care from the UK and Ireland Confidential Enquiries into maternal deaths and morbidity 2009-2012: National Perinatal Epidemiology Unit, University of Oxford; 2014.
17. Warren CE, Abuya T, Kanya L, Obare F, Njuki R, Temmerman M, et al. A cross sectional comparison of postnatal care quality in facilities participating in a maternal health voucher program versus non-voucher facilities in Kenya. BMC pregnancy and childbirth. 2015;15(1):1-14.
18. Biratu A, Haile D. Prevalence of antenatal depression and associated factors among pregnant women in Addis Ababa, Ethiopia: a cross-sectional study. Reproductive health. 2015;12(1):1-8.
19. Abebe A, Tesfaw G, Mulat H, Hibdye G. Postpartum depression and associated factors among mothers in Bahir Dar Town, Northwest Ethiopia. Annals of general psychiatry. 2019;18(1):1-8.
20. Abadiga M. Magnitude and associated factors of postpartum depression among women in Nekemte town, East Wollega zone, west Ethiopia, 2019: A community-based study. PloS one. 2019;14(11):e0224792.

21. Toru T, Chemir F, Anand S. Magnitude of postpartum depression and associated factors among women in Mizan Aman town, Bench Maji zone, Southwest Ethiopia. BMC pregnancy and childbirth. 2018;18(1):1-7.
22. Davis J, Luchters S, Holmes W. Men and maternal and newborn health: benefits, harms, challenges and potential strategies for engaging men. Melbourne, Australia: Compass: Women's and Children's Health Knowledge Hub. 2012.
23. Yargawa J, Leonardi-Bee J. Male involvement and maternal health outcomes: systematic review and meta-analysis. J Epidemiol Community Health. 2015;69(6):604-12.
24. Kerie S, Menberu M, Niguse W. Prevalence and associated factors of postpartum depression in Southwest, Ethiopia, 2017: a cross-sectional study. BMC research notes. 2018;11(1):1-7.
25. Gelaye B, Williams MA, Lemma S, Deyessa N, Bahretibeb Y, Shibre T, et al. Validity of the patient health questionnaire-9 for depression screening and diagnosis in East Africa. Psychiatry research. 2013;210(2):653-61.
26. Abiola T, Udofia O, Zakari M. Psychometric properties of the 3-item oslo social support scale among clinical students of Bayero University Kano, Nigeria. Malaysian Journal of Psychiatry. 2013;22(2):32-41.
27. Kebede AA, Cherkos EA, Taye EB, Eriku GA, Taye BT, Chanie WF. Married women's decision-making autonomy in the household and maternal and neonatal healthcare utilization and associated factors in Debretabor, northwest Ethiopia. PloS one. 2021;16(9):e0255021.
28. Stöckl H, March L, Pallitto C, Garcia-Moreno C. Intimate partner violence among adolescents and young women: prevalence and associated factors in nine countries: a cross-sectional study. BMC public health. 2014;14(1):1-14.
29. Petrosyan D, Armenian HK, Arzoumanian K. Interaction of maternal age and mode of delivery in the development of postpartum depression in Yerevan, Armenia. Journal of affective disorders. 2011;135(1-3):77-81.
30. Khalifa DS, Glavin K, Bjertness E, Lien L. Determinants of postnatal depression in Sudanese women at 3 months postpartum: a cross-sectional study. BMJ open. 2016;6(3):e009443.

31. Madeghe BA, Kimani VN, Vander Stoep A, Nicodimos S, Kumar M. Postpartum depression and infant feeding practices in a low income urban settlement in Nairobi-Kenya. BMC research notes. 2016;9(1):1-9.

32. Adama ND, Foumane P, Olen JPK, Dohbit JS, Meka ENU, Mboudou E. Prevalence and risk factors of postpartum depression in Yaounde, Cameroon. Open Journal of Obstetrics and Gynecology. 2015;5(11):608.

33. Chien L-Y, Tai C-J, Yeh M-C. Domestic decision-making power, social support, and postpartum depression symptoms among immigrant and native women in Taiwan. Nursing research. 2012;61(2):103-10.

34. Wubetu AD, Engidaw NA, Gizachew KD. Prevalence of postpartum depression and associated factors among postnatal care attendees in Debre Berhan, Ethiopia, 2018. BMC pregnancy and childbirth. 2020;20(1):1-9.

35. Ashenafi W, Mengistie B, Egata G, Berhane Y. The role of intimate partner violence victimization during pregnancy on maternal postpartum depression in Eastern Ethiopia. SAGE open medicine. 2021;9:2050312121989493.

36. Shewangzawa A, Tadesse B, Ashani T, Misgana T, Shewasinad S. Prevalence of postpartum depression and associated factors among postnatal women attending at Hiwot Fana Specialized University Hospital. Harar, East Ethiopia. 2018.

37. Gebregziabher NK, Netsereab TB, Fessaha YG, Alaza FA, Ghebrehiwet NK, Sium AH. Prevalence and associated factors of postpartum depression among postpartum mothers in central region, Eritrea: a health facility based survey. BMC public health. 2020;20(1):1-10.

38. Govender D, Naidoo S, Taylor M. Antenatal and postpartum depression: prevalence and associated risk factors among adolescents' in KwaZulu-Natal, South Africa. Depression research and treatment. 2020;2020.

39. Nur N. The effect of intimate partner violence on mental health status among women of reproductive ages: A population-based study in a middle Anatolian city. Journal of interpersonal violence. 2012;27(16):3236-51.

40. Asaye MM, Muche HA, Zelalem ED. Prevalence and predictors of postpartum depression: Northwest Ethiopia. Psychiatry Journal. 2020;2020.

41. Shitu S, Geda B, Dheresa M. Postpartum depression and associated factors among mothers who gave birth in the last twelve months in Ankesha district, Awi zone, North West Ethiopia. BMC pregnancy and childbirth. 2019;19(1):1-9.

42. Liu S, Yan Y, Gao X, Xiang S, Sha T, Zeng G, et al. Risk factors for postpartum depression among Chinese women: path model analysis. BMC pregnancy and childbirth. 2017;17(1):1-7.

43. Salsabilla DA, Prasetya H, Murti B. The Effect of Unplanned Pregnancy on Postpartum Depression: A Meta-Analysis. Journal of Maternal and Child Health. 2020;5(5):500-13.

44. Barton K, Redshaw M, Quigley MA, Carson C. Unplanned pregnancy and subsequent psychological distress in partnered women: a cross-sectional study of the role of relationship quality and wider social support. BMC pregnancy and childbirth. 2017;17(1):1-9.

45. Centers for Disease C, Prevention. Prevalence of self-reported postpartum depressive symptoms--17 states, 2004-2005. MMWR Morbidity and mortality weekly report. 2008;57(14):361-6.

46. Mahfouz E, Mohammed E, Alkilany S, Rahman TA. Impact of household food insecurity on maternal mental health in Egypt. Eastern Mediterranean Health Journal. 2021;27(4).

47. Abimana MC, Karangwa E, Hakizimana I, Kirk CM, Beck K, Miller AC, et al. Assessing factors associated with poor maternal mental health among mothers of children born small and sick at 24–47 months in rural Rwanda. BMC pregnancy and childbirth. 2020;20(1):1-12.

48. Osamor PE, Grady C. Women's autonomy in health care decision-making in developing countries: a synthesis of the literature. International journal of women's health. 2016;8:191.

49. Balabanova ES. Women's economic dependency: Nature, causes, and consequences. Sociological research. 2007;46(2):5-21.

50. Ampim GA. Men's Involvement in Maternal Healthcare in Accra, Ghana. From Household to Delivery Room: The University of Bergen; 2013.

51. Abbasi S, Chuang CH, Dagher R, Zhu J, Kjerulff K. Unintended pregnancy and postpartum depression among first-time mothers. Journal of women's health. 2013;22(5):412-6.

Questionnaire

Click here to access/download

Supporting Information

English version of the questionnaire.docx

Click here to access/download
Supporting Information
SPSS data.sav
