# Supplementary material for: Low husband involvement in maternal and child health services and intimate partner violence increases the odds of postpartum depression in northwest Ethiopia: A community-based study
Source: PLoS One. 2022 Oct 26;17(10):e0276809. doi: 10.1371/journal.pone.0276809 (PMC9604988; doi:10.1371/journal.pone.0276809)
Supplement: Attachment — Submitted filename: PONE-D-21-35177.docx [file pone.0276809.s004.docx]

Overall, the manuscript is good and has been organized well. I have a few of comments to improve the manuscript as follows:

1.Abstract: the abstract is good and concise. However, I noticed you didn’t write anything about discussion that you have mentioned in the manuscript.

-in the result of abstract section, you mentioned about MNCH and in the conclusion you have written, ‘maternal and child health’. Two are the same things. please keep consistency.

2. Introduction: You could add some reference on IPV in national and local context that could strengthen your argument in the paper.

- you mentioned you did not compare the PPD screening tool with national guideline? Why? What were the gap in the national guideline? Did you explore or analyze it?

3. Method: In the method section, you said that you choose sample from mothers who were first time mother and who had experienced multiple pregnancy. Can you mention how many Primi mother you have interviewed who were suffering from depression?

-can you explain clearly about lottery method or any reference?

-Two data collector completed 757 interview while non response rate was 10%. They collected data within a single month. How many interview they conducted per day? How the data quality was checked? Can you explain it a bit more?

4. Result:

You have mentioned women’s socio-cultural aspects were linked to the postpartum depression. How religious background linked to depression? Would you please explain.

- 92.8% pregnancy was planned and had family support during pregnancy that you mentioned. If so how this variable was selected as the cause of depression?
- Family income is another factor you mentioned. I am saying, poverty is a common reason of mental stress in any situation. Why did you particularly mention it as a cause of postpartum depression? Please explain.
- You have focused on IPV more than any other variables you mentioned in the manuscript. But IPV itself a big area which leads to worse mental health situation. I think you did not mention all the component of IPV that leads to postpartum depression. I encourage you to recheck your data and dig down the path.

5. you have written well, but it would be stronger if you could add more reference on this issues you have explored in your research.

-What about the national data about postpartum depression. Does it support your data? Did you check other study in Ethiopia’s other regions that support your data?

-What about the policy implication? How your data recommend to improve mental health situation of the women in the country? Please explain
