# Supplementary material for: Low husband involvement in maternal and child health services and intimate partner violence increases the odds of postpartum depression in northwest Ethiopia: A community-based study
Source: PLoS One. 2022 Oct 26;17(10):e0276809. doi: 10.1371/journal.pone.0276809 (PMC9604988; doi:10.1371/journal.pone.0276809)
Supplement: Attachment — Submitted filename: Authors response to reviewers.docx [file pone.0276809.s005.docx]

**Date: June 4/ 2022**

**Point by point response to reviewers comment**

**Manuscript title:** Low husband involvement in maternal and child health services and intimate partner violence increases the odds of postpartum depression in northwest Ethiopia: a community-based study

**Manuscript Ref:** Submission ID PONE-D-21-35177

Dear reviewers, thank you for the most important issues you raised for the betterment of our manuscript. Below are the responses to the comments and concerns you raised. In addition, we incorporated your comments and suggestions in the revised manuscript.

**Response to reviewer’s comments**

Reviewer Comments:

**#Reviewer 1**

**Introduction**

1. It can lead to suicide, but not in all or even most cases, so you shouldn’t say it will.

**Author’s response:** Thank you dear reviewer for your insightful comments. It has been corrected in the revised manuscript

2. Male involvement? Sounds clearer

**Author’s response:** Dear reviewer, thank you. It was typing error and corrected in the revised manuscript.

3. Do you mean poor maternal health in general or only poor maternal mental health here?

**Author’s response:** Dear reviewer, thank you for the comments. We have stated that the effect of low husband/partner involvement in MNCH care service on maternal health in general.

4. Which PPD screening tool? I am aware of more than one, so please specify which you are talking about here.

**Author’s response:** Thank you dear reviewer. We have specified it in the revised manuscript. Please see the revised manuscript.

**Method and materials**

5. How are you defining reproductive age?

**Author’s response:** Dear reviewer, thank you for your comment. Reproductive age is defined as women aged from 15-49 years. It has been defined in the revised manuscript.

6. The study population included all women who gave birth **in the last year** and who resided in the city for at least 6 months in the selected kebeles during the data collection period. Put actual dates.

**Author’s response:** Dear reviewer, thank you. It has been added in the revised manuscript.

7. The sample size was determined by using the single population proportion formula by considering the following assumptions: The proportion of postpartum depression 33.82% (26). Where did this specific number come from?

**Author’s response:** Thank you dear reviewer. We used the proportion of postpartum depression from a previous study that was done in southwest Ethiopia. It has been specified in the revised manuscript. Please see the revised manuscript.

8. On variables of the study: Having ANC visit. Did you only look at one ANC visit? Or the recommended number?

**Author’s response:** Thank you dear reviewer for your important comment. In our study, a postpartum woman was categorized as having an ANC visit if she had at least one ANC visit. Sorry, we missed it in the descriptive part about the number of ANC visits. It has been included in the revised manuscript (Table 2).

9. Household decision-making power: The ability of women to act independently on the household activities including their health, children’s health, freedom of movement, and control over finance, decides on large household purchases. This is confusingly worded.

**Author’s response:** Thank you dear reviewer. It has been corrected in the revised manuscript. Please look at the revised manuscript.

10. Thus, based on the summative score of variables designed to assess household decision-making power women who were answered above the mean value were considered as having higher decision-making power. What was that value? What was the range? I am confused about the scores on this.

**Author’s response:** Dear reviewer thank you for important comment. It has been corrected in the revised manuscript.

11. Husband/partner involvement in MNCH. What was that value? What was the range? I am confused about the scores on this.

**Author’s response:** Dear reviewer thank you for important comment. It has been corrected in the revised manuscript.

12. Who were the interviewers?

**Author’s response:** Dear reviewer thank you. We mentioned that the data collectors were BSc midwifes.

**Result**

13. The majority, 773 (97.4%) of study participants had at least one **ANC follow up**. The use of the term ANC follow up is confusing as a follow up is usually an appointment after a medical event, and ANC is before the birth.

**Author’s response:** Dear reviewer thank you. We tried to correct as ANC visit in the revised manuscript.

14. Pregnancy was. Correct as pregnancies were

**Author’s response:** Dear reviewer, thank you. It has been corrected in the revised manuscript.

15. The magnitude of postpartum depression among women who gave birth in the last one year was 17.25% (95% CI: 14.5, 20.2). It that prevalence?

**Author’s response:** Thank you very much dear reviewer. Yes, it is prevalence of PPD. It has been corrected in the revised manuscript.

16. The study also **declared** that there was a significant association between IPV and postpartum depression. Found? a study can’t really declare something

**Author’s response:** Thank you dear reviewer for your important comment. We tried to correct it in the revised manuscript.

17. Table 2. ANC follow up. Again, use of the term follow up when talking about ANC is not appropriate

**Author’s response:** Thank you dear reviewer for your important comment. We tried to correct it in the revised manuscript.

18. Table 2. How were you defining medical disorder? That could mean many different things.

**Author’s response:** Dear reviewer, thank you for your comment. We mean that medical illness and we tried to define it in the revised manuscript in the operational definition section. Please see the revised manuscript.

**Discussion**

19. A large portion of this section focuses on comparing the prevalence of PPD in this study to other studies. Based on the beginning of this paper, determining the prevalence of PPD isn’t the main purpose of the study, so talking about that for so long isn’t adding anything to the discussion.

**Author’s response:** Thank you. This study aimed to assess the prevalence and associated factors of postpartum depression, and we modified the title based on our findings to give emphasis. But if the modification of the title is not necessary, we can remove it.

20. You aren’t talking about what this study is actually adding to the literature. What new information did we get from this study? And what can we do with that information?

**Author’s response:** Thank you, dear reviewer. In this study, we tried to assess the prevalence of PPD and identify which factors were associated with it in our study setting. As a new finding, husband/partner involvement in MNCH care services was significantly associated with higher odds of PPD. Based on this finding/information, we recommend that the government prioritize husband involvement in MNCH care services in order to avert this public health issue. Moreover, based on our findings, we tried to clarify our recommendations in the revised manuscript.

21. This community-based cross-sectional study assessed postpartum depression and associated factors among women who gave birth **in the last one year** in Gondar city, northwest Ethiopia, 2021. Again, use specific dates.

**Author’s response:** Dear reviewer, thank you. We used the specific date in the revised manuscript.

22. I assume you are still comparing PPD prevalence at this point? This study had many findings, so be specific.

**Author’s response:** Dear reviewer, thank you for your important comment. As we said earlier, our first objective was to determine the prevalence of postpartum depression. The second objective was to identify factors that are significantly associated with PPD. Based on this, we tried to address all the objectives. This was our reason for comparing our study findings with other studies that were conducted previously.

23. Because postpartum depression is most common in the first 6 weeks after child birth (21). It is incomplete sentence.

**Author’s response:** Dear reviewer, thank you. We tried to complete this sentence in the revised manuscript. Please see the revised manuscript.

24. This could be explained by being supported by a husband or partner will build a higher sense of support to the women. This sentence is confusingly worded.

**Author’s response:** Thank you dear reviewer for your important comment. We tried to make it clear in the revised manuscript. Please see the revised manuscript.

25. This is because men are influential in making health care decisions that may make the women feeling of under estimated often leading to mental health problems. Also confusingly worded.

**Author’s response:** Thank you dear reviewer. It has been corrected in the revised manuscript.

26. There are additional study limitations. For example, the use of interviewer-delivered surveys can lead to issues like social desirability bias or interviewer bias.

**Author’s response:** Thank you very much, dear reviewer for your valuable suggestions. We tried to incorporate your suggestion as well as other limitations of this study in the revised manuscript.

27. What additional research is needed? Are there any specific activities you would recommend?

**Author’s response:** Thank you, dear reviewer for your comment. For future researcher, we tried to write our recommendations in the revised manuscript.

28. You mentioned that a screening tool for PPD wasn’t included in the guidelines for care in Ethiopia. Would you recommend it is? If so, how do you recommend that be done? I am left at the end of this study think “so what” because it isn’t clear what exactly you want done with this information or how it can be built on.

**Author’s response:** Thank you, dear reviewer for your comment. We authors recommend the inclusion of a PPD screening tool in antenatal and postnatal care service guidelines in order to early identify those women who are at risk and tackle postpartum depression.

**#Reviewer 2**

**Abstract:**

1. The abstract is good and concise. However, I noticed you didn’t write anything about discussion that you have mentioned in the manuscript.

**Author’s response:** Thank you dear reviewer for your important comment. As an abstract writing format, it includes background, methods, results, and conclusions. Based on this format, there was no room to write a discussion in the abstract. So this was our reason for not including the discussion in the abstract section.

2. In the result of abstract section, you mentioned about MNCH and in the conclusion you have written, ‘maternal and child health’. Two are the same things. Please keep consistency.

**Author’s response:** Dear reviewer thank you for your suggestions. It has been corrected in the revised manuscript.

**Introduction:**

3. You could add some reference on IPV in national and local context that could strengthen your argument in the paper.

**Author’s response:** Thank you dear reviewer for your important comment. We tried to add information about IPV in national and local context in the revised manuscript.

4. You mentioned you did not compare the PPD screening tool with national guideline? Why? What were the gap in the national guideline? Did you explore or analyze it?

**Author’s response:** Dear reviewer, thank you for your comment. We would like to say sorry for the inconvenience. We mentioned this sentence…" However, the PPD screening tool is not incorporated into modern postnatal care guidelines in Ethiopia" in our manuscript in the introduction section. But we did not state that we did not compare the PPD screening tool with national guidelines in our manuscript.

**Method:**

5. In the method section, you said that you choose sample from mothers who were first time mother and who had experienced multiple pregnancy. Can you mention how many Primi mother you have interviewed who were suffering from depression?

**Author’s response:** Dear reviewer thank you for your comment. Sorry for the inconvenience. We tried to explain that we chose samples from women who gave birth in the last year and who resided in the city for at least 6 months in the selected kebeles during the data collection period, but we did not say we chose samples from mothers who were first-time mothers and who had experienced multiple pregnancies. Based on our finding, among the total primi mothers, 42 (13.2%) of women experienced PPD.

6. Can you explain clearly about lottery method or any reference?

**Author’s response:** Thank you, dear reviewer for your important comment. As a rule of thumb, considering the feasibility and the information gained the scholars suggested that it is better to have a sample at least in 20%–50% of the kebeles. Therefore, to achieve the probability sampling method, we tried to select six kebeles (27%) in Gondar city by lottery method to gain our sample size. Besides, all kebeles in our study area had an equal chance. Therefore, we can generalize our findings to the source populations.

7. Two data collector completed 757 interview while non response rate was 10%. They collected data within a single month. How many interview they conducted per day?

**Author’s response:** Thank you, dear reviewer. Sorry for the inconvenience. In our manuscript we have stated that six BSc midwives for data collection and two MSc midwife supervisors were recruited.

8. How the data quality was checked? Can you explain it a bit more?

**Author’s response:** Dear reviewer, thank you for your comment. We tried to assure the data quality by providing training for both the data collectors and supervisors before the actual data collection. We conducted a pretest and the necessary corrections were made. In the actual data collection period, the completeness of the questionnaire was checked by the supervisors daily. We tried to verify in the revised manuscript. Please see the revised manuscript.

**Result:**

9. You have mentioned women’s socio-cultural aspects were linked to the postpartum depression. How religious background linked to depression? Would you please explain?

**Author’s response:** Thank you dear reviewer for your important comment. In our study, the data collection tool was developed by reviewing the literature. So, based on the developed data collection tool, we tried to assess the association of religion with PPD. Unfortunately, there is no association. This religion may have a link with PPD, as the strength of religious commitment can be positively or negatively associated with PPD.

10. 92.8% pregnancy was planned and had family support during pregnancy that you mentioned. If so how this variable was selected as the cause of depression?

**Author’s response:** Thank you dear reviewer for your important comment. In our study, 85.4% and 92.8% of pregnancies were planned and had family support during pregnancy, respectively. The variables were selected based on a review of previous literature and incorporated into our study data collection tool to test whether they have a statistically significant association with PPD or not by using a logistic regression analysis model. As a result, planned pregnancy has a statistically significant positive association with PPD. This are some literatures:

<https://link.springer.com/article/10.1186/s12991-019-0244-4>, <https://journals.plos.org/plosone/article?id=10.1371/journal.pone.0224792>, <https://www.liebertpub.com/doi/abs/10.1089/jwh.2012.3926>

11. Family income is another factor you mentioned. I am saying, poverty is a common reason of mental stress in any situation. Why did you particularly mention it as a cause of postpartum depression? Please explain.

**Author’s response:** Thank you dear reviewer for your valuable comment. Obviously, poverty is a reason for mental health problems. But in our study, we included family income as an independent variable because our interest was to show to what extent poverty affects women's mental health (postpartum depression).

12. You have focused on IPV more than any other variables you mentioned in the manuscript. But IPV itself a big area which leads to worse mental health situation. I think you did not mention all the component of IPV that leads to postpartum depression. I encourage you to recheck your data and dig down the path.

**Author’s response:** Thank you, dear reviewer for your valuable comment. This study aimed to assess the prevalence and associated factors of PPD but, based on our findings, we modified the title to give emphasis. To strengthen our study, we tried to include some information about IPV and husband/partner involvement in MNCH care services in the introduction section. Frankly speaking, IPV by itself is a big area, but we were able to include all domains of IPV (physical, sexual, and emotional violence) with their specific measurement tool. Besides, in this study, we tried to discuss each finding in detail.

**Discussion**

13. You have written well, but it would be stronger if you could add more reference on this issues you have explored in your research.

**Author’s response:** Dear reviewer, thank you for your comment. We tried to add more reference in the revised manuscript.

14. What about the national data about postpartum depression. Does it support your data? **Author’s response:** Thank you dear reviewer. The national data showed that the prevalence of postpartum depression was 21.55 and factors that revealed from the national study supports our findings.

[https://www.sciencedirect.com/science/article/abs/pii/S0165032720329839#](https://www.sciencedirect.com/science/article/abs/pii/S0165032720329839)!

15. Did you check other study in Ethiopia’s other regions that support your data?

**Author’s response:** Thank you dear reviewer for your important comment. We checked and used studies done in other regions of Ethiopia in our discussion. Such as a study done in Southwest and Eastern Ethiopia that supports our findings. This are the link;

<https://link.springer.com/article/10.1186/s12884-018-2072-y>

<https://journals.sagepub.com/doi/abs/10.1177/2050312121989493>

16. What about the policy implication?

**Author’s response:** Dear reviewer thank you very much for your insightful comments. Based on our study finding we tried to include its policy implication in the revised manuscript. Please see the revised manuscript.

17. How your data recommend to improve mental health situation of the women in the country? Please explain

**Author’s response:** Thank you, dear reviewer for your valuable comment. Based on our data, we have stated some recommendations for early identification and treatment of PPD, but we tried to include additional recommendations in the revised manuscript to improve mental health problems. Please see the revised manuscript.
