# Supplementary material for: Low husband involvement in maternal and child health services and intimate partner violence increases the odds of postpartum depression in northwest Ethiopia: A community-based study
Source: PLoS One. 2022 Oct 26;17(10):e0276809. doi: 10.1371/journal.pone.0276809 (PMC9604988; doi:10.1371/journal.pone.0276809)
Supplement: Attachment — Submitted filename: Review report_D-21-35177_R1.docx [file pone.0276809.s006.docx]

Review report of D-21-35177:

I would like to thank to the authors who addressed majority of the comments on the issues I raised during my first review. This is an important manuscript having data with mental health issues.

However, still I didn’t understand the lottery method theoretically. If possible, explain it or provide a reference which will help readers to understand.

Wish you good luck!
