# Supplementary material for: Low husband involvement in maternal and child health services and intimate partner violence increases the odds of postpartum depression in northwest Ethiopia: A community-based study
Source: PLoS One. 2022 Oct 26;17(10):e0276809. doi: 10.1371/journal.pone.0276809 (PMC9604988; doi:10.1371/journal.pone.0276809)
Supplement: Attachment — Submitted filename: Authors response.docx [file pone.0276809.s007.docx]

**Date: August 31/ 2022**

**Point by point response to reviewers comment**

**Manuscript title:** Low husband involvement in maternal and child health services and intimate partner violence increases the odds of postpartum depression in northwest Ethiopia: a community-based study.

**Manuscript Ref:** Submission ID PONE-D-21-35177R1

We are very grateful to both the editor and reviewers for your comments and concerns for the betterment of our manuscript. Appreciating your effort and valuable comments, we have provided possible reflections on the raised concerns and questions. Kindly find our responses here. In addition, we incorporated your comments and suggestions in the revised manuscript.

**Response to reviewer’s comments**

Reviewer Comments:

**#Reviewer 1**

1. Because of the timing of this study, it is important to acknowledge the potential impacts of the COVID 19 pandemic and the conflict in Tigray, as it isn’t too far from Gondar City, on the mental health of the women included in the study. Evidence collected around the world has shown an increase in IPV during the pandemic, and this would likely have an impact on PPD. Also the pandemic itself has had a massive effect on mental health directly. There is also evidence that living in or near a conflict zone has dramatic impacts on mental health, further exacerbating the chances of PPD. Your study didn’t collect data related to this, but at least acknowledging the potential impacts and that you are comparing your findings to studies pre-COVID could skew the data.

**Author’s response:** Thank you dear reviewer for your insightful comment. It has been considered in the revised manuscript.

2. You don’t really “have” IPV, it isn’t a disease, and it is something that you experience or is done to you. The language around this topic should be adjusted.

**Author’s response:** Dear reviewer, thank you for your important comment. It has been corrected in the revised manuscript. Please look at the revised manuscript.

3. There still needs to be more information in the conclusion/discussion about what the outcomes from this study should be. You talk about policymakers and researchers a little, but what about practitioners who will be working directly with these patients? What do you recommend as to screening? I'm still not clear on what your recommendations are regarding regular screening for PPD.

**Author’s response:** Thank you dear reviewer for your important comment. We have tried to add further recommendations. Please look at the revised manuscript.

**#Reviewer 2**

1. I would like to thank to the authors who addressed majority of the comments on the issues I raised during my first review. This is an important manuscript having data with mental health issues. However, still I didn’t understand the lottery method theoretically. If possible, explain it or provide a reference which will help readers to understand.

**Author’s response:** Dear reviewer, thank you for your invaluable contribution to the improvement of our manuscript. We simply meant that simple random sampling method. The lottery method is one type of simple random sampling technique, and we used it by assigning a number to each kebele (the smallest administrative unit), after which numbers are selected at random.
