# Supplementary material for: Low husband involvement in maternal and child health services and intimate partner violence increases the odds of postpartum depression in northwest Ethiopia: A community-based study
Source: PLoS One. 2022 Oct 26;17(10):e0276809. doi: 10.1371/journal.pone.0276809 (PMC9604988; doi:10.1371/journal.pone.0276809)
Supplement: Attachment — Submitted filename: Reviewer comments_PONE-D-21-35177_R2.docx [file pone.0276809.s008.docx]

Reviewer comments:

PONE-D-21-35177_R2

Low husband involvement in maternal and child health services and intimate partner violence increases the odds of postpartum depression in northwest Ethiopia: a community-based study

Comments: I think the author has clarified the questions I raised.

Thank you and wish you very good luck!
